# Explorative Study on Isolation and Characterization of a *Microviridae* G4 Bacteriophage, EMCL318, against Multi-Drug-resistant *Escherichia coli* 15-318

**DOI:** 10.3390/antibiotics7040092

**Published:** 2018-10-26

**Authors:** Soumya Ghosh, Emma Persad, Ting-Yun Shiue, Cindy Lam, Afsana Islam, Lauren G. Mascibroda, Michael B. Sherman, Thomas Smith, Naowarat Cheeptham

**Affiliations:** 1 Department of Biological Sciences, Faculty of Science, Thompson Rivers University, Kamloops, BC V2C 0C8, Canada; Soumyaghosh@yahoo.com (S.G.); emmapersad@gmail.com (E.P.); yvonnes518@gmail.com (T.-Y.S.); syndilam@gmail.com (C.L.); afsana.islam.tonu@gmail.com (A.I.); 2 Medical School, Karl Landsteiner Private University for Health Sciences, Dr. Karl Dorrek Strasse 30, A-3500 Krems an der Donau, Austria; 3 Fort St. John Hospital Laboratory, Fort St. John, BC V1J 2A4, Canada; 4 Department of Biochemistry and Molecular Biology, University of Texas Medical Branch, 301 University Blvd, 5.104D Basic Science Building, Galveston, TX 77555, USA; lgmascib@UTMB.EDU (L.G.M.); mbsherma@utmb.edu (M.B.S.)

**Keywords:** Multi-drug-resistant organisms, bacteriophages, phage therapy, alternative therapeutic measures

## Abstract

Bacteriophages screened and isolated from sewage water samples exhibited antibacterial activities against multi-drug-resistant *Escherichia coli* strains. Five different water samples from Canadian habitats such as Kamloops Wastewater Treatment Center, Domtar, the Pacific Ocean, Bisaro Anima Cave, and alkali ponds, were used in this study. Four *Enterobacteriaceae* strains including one non-resistant and three clinical multi-drug *Escherichia coli* strains (*E. coli* 15-102, *E. coli* 15-124, and *E. coli* 15-318) were selected as target bacteria to screen for the bacteriophages from these collected water samples. Seeded agar assay technique was implemented for the screening. It was found that only sewage water sample exhibited a significant number of plaques count with the *E. coli* 15-318 (1.82 × 10^2^ plaques/plate) cells in comparison to *E. coli* non-resistant strain K12 (8 plaques/plate). The phage did not produce plaques in the *E. coli* 15-124 and *E. coli* 15-102 strains. The bacteriophage, designated EMCL318, was isolated, purified, characterized, and identified to belong to the G4 species of the Family *Microviridae*, GenBank accession number MG563770. This is an explorative study conducted in order to reveal the viruses as alternative potentials to fight against emerging and existing multi-drug-resistant infectious diseases.

## 1. Introduction

The extensive overuse of antibiotics in humans, veterinary, and agricultural practices over the decades and their continuous release as effluents from the urban wastewater treatment plants in the environment and from untreated effluent of pharmaceutical industry [1,2,3,4] pose a significant public health threat [5]. High levels of antibiotics in the environment leads to rapid emergence of antibiotic-resistant genes (ARGs) and bacteria (ARB), reducing the therapeutic capacity of these existing commercially available antibiotics [6,7]. These multi-drug-resistant organisms (MDROs) exhibit resistance to more than one antimicrobial agents [5,6]. In most of the cases, the current drugs to treat MDRO infections are expensive and have toxicity issues [6]. For example, the infections caused by *Pseudomonas aeruginosa* and *Acinetobacter baumannii* are sometimes resistant to all antibiotics and can be fatal to immunocompromised individuals [8,9].

Though multidrug resistant (MDR) strains were first observed among enteric bacteria, in particular coliforms and enterococci, it was found that chief among antibiotic-resistant bacteria are Gram-positive methicillin-resistant *Staphylococcus aureus* (MRSA) and vancomycin-resistant *Enterococcus* spp. with several other Gram-negative bacteria that are resistant to fluoroquinolones [7]. These resistant bacterial strains have been identified to contain enzymes such as carbapenemases and extended spectrum β-lactamases (ESBL), causing broad resistances for treatment [10]. The antibiotic-resistant encoding genes in bacteria are often located on the plasmids of the MDR strains and are transferred with high frequency during bacterial conjugation [10,11]. Infections from bacteria which possess these enzymes render antibiotic treatments highly unsuccessful, illustrating the importance of development of an alternative treatment method such as phage therapy. In recent years, *E. coli* has emerged as one of the major MDR strains that has become a global concern [12]. For instance, the *E. coli* sequence type (ST) 131 has been consistently reported in association with urinary tract infections (UTI) and bacteremia [13]. In another study, a New Delhi metallo-enzyme (NDM) carbapenase variant of multidrug-resistant *E*. *coli* ST648 isolated from perinium and throat of a patient from UK has conferred resistance to all β-lactams [14].

Phage therapy is the application of phage that targets and kills specific pathogenic bacteria [15]. Phages were first discovered by British microbiologist Felix Twort in 1915, and later by French-Canadian microbiologist Felix d’Hérelle in 1917. Although Twort did not pursue his discovery, d’Hérelle investigated the nature and mechanism of phages as therapeutic agents, and established phage therapy centers in the U.S., France, and Soviet Georgia [16]. He first screened phages from French troops with severe hemorrhagic dysentery in July 1915, where bacterium-free filtrates of patient’s fecal samples were incubated with isolated *Shigella* strains from the patients. Clear areas (plaques) were observed and it was concluded that these viruses are capable of parasitizing bacteria [16]. These phages were later used in phage therapy trials in 1919 on hospital patients with severe dysentery [17]. The use of bacteriophages as therapeutic agents was later used extensively during World War II, particularly by Soviet doctors to treat wound infections of troops on the battlefield. However, the discovery of penicillin in 1928 caused a sharp decline in phage research in the West, which chose to prioritize treatment of bacterial infections with antibiotics, leaving only the former Soviet Union countries still developing and utilizing phage therapy [15].

This study emphasizes explorative research on screening for, isolation and characterization of a bacteriophage found in Kamloops Wastewater Treatment Centre (British Columbia, Canada) that demonstrated antibacterial/lytic activity against clinical multidrug resistant *E. coli* strain 15-318. The investigation identified a phage that possesses anti-*E. coli* 15-318 activity, this phage was designated as EMCL318 and was isolated, purified and characterized. The study is an additional contribution to determining a possible therapeutic measure against antibiotic-resistant infections.

## 2. Results

### 2.1. Bacteriophages Isolation

**Figure 1:**
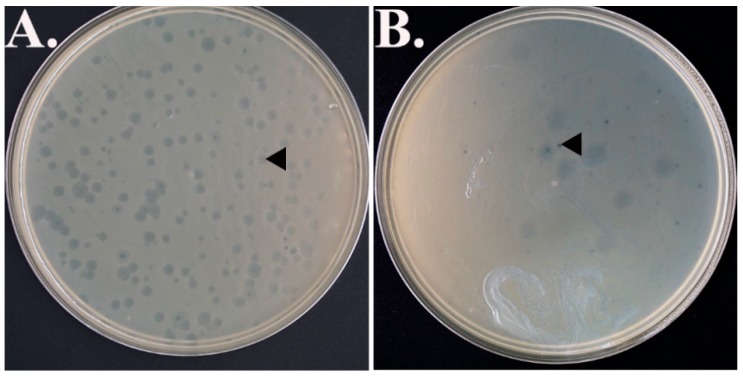
Plaques formation (as indicated with the black arrow heads) on (**A**) *E*. *coli* 15-318 strain, (**B**) Non-resistant *E*. *coli* strain K12.

The sewage water sample collected from the Kamloops Wastewater Treatment Center exhibited abundant plaque formation (1.82 × 10^2^ plaques/plate) against the *E*. *coli* 15-318 strain while tracer amounts of plaques were identified against regular non-resistant *E*. *coli* strain K12 (Figure 1). However, no plaques were identified from the water samples obtained from Domtar, the Pacific Ocean, Bisaro Anima Cave, and alkali ponds.

### 2.2. Bacteriophage Purification and Identification

After four rounds of plaque purification, attempts were made to amplify the titer of phage in liquid culture. However, the titer decreased when grown in *E. coli* 15-318, at 37 °C. As with many phages, the best yield comes with a balance of bacterial growth with phage production. Therefore, the incubation was moved to room temperature (24 °C) and the duration was increased to 48 h, and this greatly improved the phage yield. Since it was not known whether the phage contained membranes, only detergent-free purification methods were employed. The cells were removed by centrifugation and the clarified supernatant was concentrated via a tangential flow concentrator with a 300 kD cutoff. The exudate did not have any measurable amount of phage. The concentrate, 100–200 mL, was then pelleted by centrifugation for 2 h at 45,000 rpm in a 50.2Ti rotor (184,000× *g*). The pellets were re-suspended and purified using a 7.5–45% sucrose gradient. A faint band was observed 2/3 of the way from the top, as determined using an ISCO fractionator. Plaque assays confirmed the presence of the phage and the most active fractions were pooled, dialyzed against PBS, and examined by SDS-PAGE. Two of the major bands from the SDS-PAGE were isolated and submitted for mass spectrometry analysis. The major band was identified as being protein F from G4 phage (expectation score of 6.3 × 10^−35^). The second band was identified as being ompC from *E. coli*.

**Figure 2:**
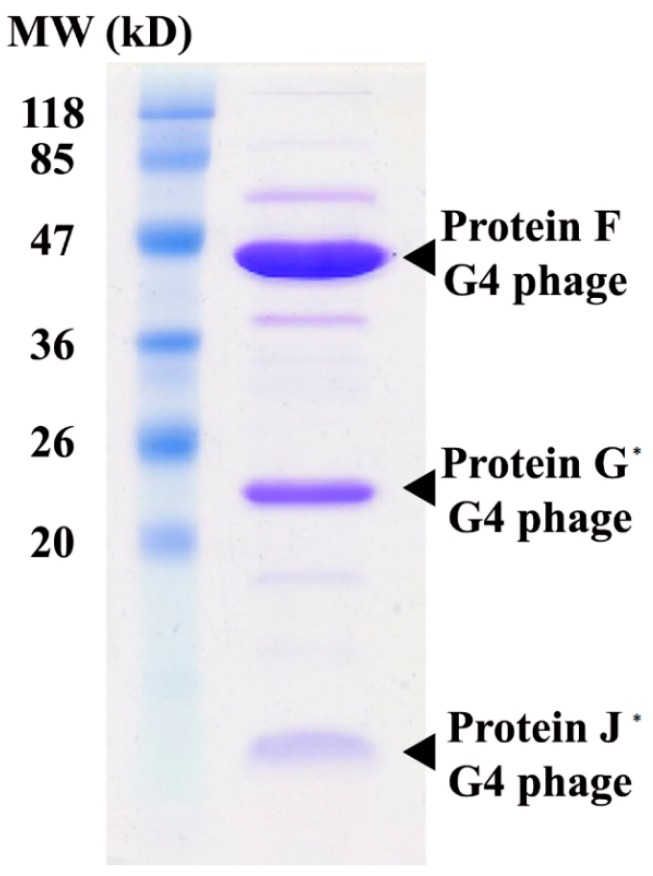
SDS-PAGE of the purified phage. The major band was identified as G4 F protein using mass spectrometry. The J and G proteins are denoted with asterisks (*) since they are only tentatively identified from the molecular weight.

With the phage being identified as a member of the G4 clade, several changes were made to the purification protocol. To the growth medium (LB), 25 mM MgCl_2_ was added to improve lysis. The addition of MgCl_2_ to the plaque assay made for clearer and larger plaques. With the addition of the MgCl_2_ to the growth media, the titer of the virus was typically ~10^7^ pfu mL^−1^ after 24 h and ~10^8^ pfu mL^−1^ after 48 h. Since the phage was identified as G4, the protocol was adjusted to remove the membrane-associated contaminants. After the clarified cell culture was concentrated with the tangential flow concentrator, EDTA and N-lauroylsarcosine was added to yield concentrations of 25 mM and 1% final concentrations, respectively. After the 2-h, 45,000 rpm centrifugation, the pellets were demonstrably smaller with the addition of the detergent and the amount of membrane-associated ompC was also significantly diminished. The pellets were re-suspended in PBS and then placed on a 7.5–45% sucrose gradient. Again, a clear band was not visible after centrifugation, most likely due to the high background of the contaminants. Nevertheless, the gradients were fractionated according to volume and each fraction was tested for infectivity. The middle portion of the gradient was found to contain the majority of the virus and those fractions were pooled and dialyzed against PBS. After dialysis, the sample was concentrated via centrifugation at 50,000 rpm in a 70.1Ti rotor (171,000× *g*). The final virus sample was analyzed by SDS-PAGE as shown in Figure 2. The F protein (shell protein, ~48 kD) was identified from the previous mass spectroscopy analysis. The next two major bands are tentatively identified as G protein (spike protein, ~20 kD) and J protein (small core protein, ~3 kD). Both proteins are expected to have a stoichiometry of 60/capsid and should appear to be major bands on the SDS-PAGE.

Further confirmation of the phage belonging to G4 clade came from negative stain electron microscopy (data not shown). The sample was a mixture of different particles, but the majority had distinct protrusions at the icosahedral 5-fold axes typical of ΦX174 [18] and G4 phages [19]. A few of the particles are smoother with a larger radius and could be various DNA packaging intermediates as previously described [20].

### 2.3. Cryo-EM Image Reconstruction

In order to verify that this phage is a member of the *Microviridae* family, the cryo-EM structure of the purified virus was determined. Twenty-six images were collected at the UTMB Sealy Center for Structural Biology at defocus values 0.75–5.7 µm. In total, 2644 particles images were selected and used for subsequent image reconstructions. The atomic structure of G4 [19] was used as an initial model. The final resolution using the gold standard method was estimated to be ~9.3 Å.

**Figure 3:**
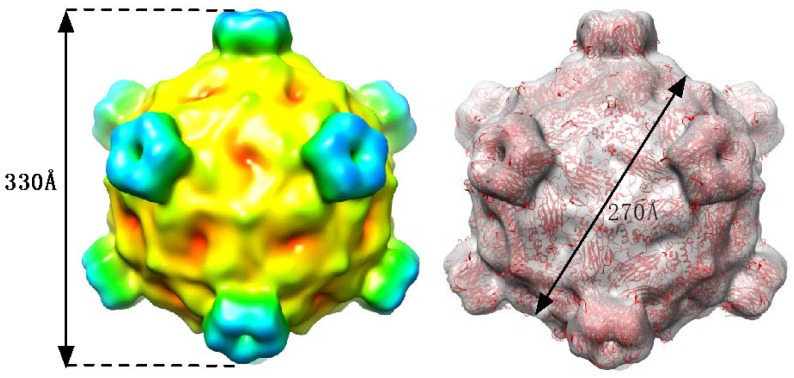
Cryo-EM structure of the purified phage. On the left is the 9.3 Å image reconstruction of the phage with the surface colored according to the radius. On the right is the image reconstruction with the structure of G4 [19] shown in the electron density envelope.

Shown in Figure 3 is the image reconstruction of the purified phage. From this structure, it is very clear that this phage belongs to the *Microviridae* family. As with other members of this family, there are marked protrusions at the icosahedral 5-fold axes. The diameter of the particles from the tip to tip of these protrusions is ~330 Å with the diameter across the base of the particle being ~270 Å. From the structure of G4 [19], the spikes are composed of the G protein and the shell is comprised of the F protein. Also shown in this figure is how well the atomic structure of G4 fits within the cryo-EM envelope. From this structure and the mass spectrometry results, this phage is a member of the *Enterobacteriaceae* phage G4 species. These two results aided in the design of sequencing primers.

### 2.4. Sequencing Results

The DNA sequence for the phage was 5,489 bases long and the accession number in GenBank is MG563770. A simple BLAST search found it to be 99% identical (79 base differences) to Coliphage WA14 (GenBank: DQ079909.1, NCBI, Bethesda, MD, USA) isolated from Pullman, Washington [21]. The 1% base differences only resulted a small number of amino acid changes in the gene products; 1 change in A/A*, 1 in C, 2 in D, 3 in F, 1 in G, and 1 in H. Proteins B, E, J, and K were identical. The next highest score came with coliphage ID18 with an identity of 98%. ID18 was isolated from Moscow, Idaho [21].

**Figure 4:**
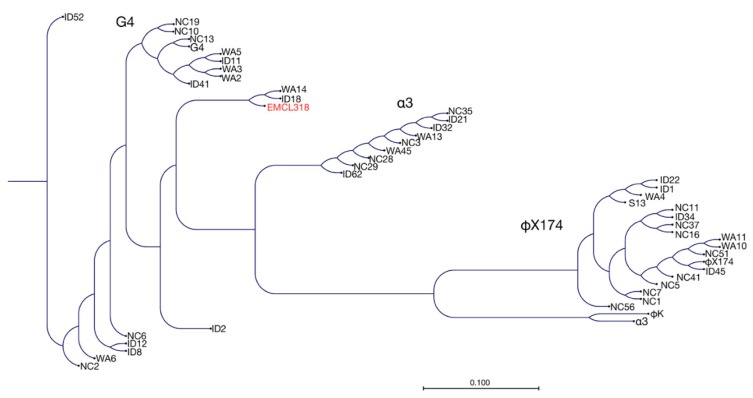
Phylogenetic relatedness among F proteins derived from different *Microviridae*. Neighbor-joining tree based on the deduced amino acid sequences from the F proteins using the CLC Sequence Viewer 7 platform (selected alignment by CanadianF alignment). The bootstrap test was performed with 100 replicates. The evolutionary distances were computed using the Jukes-Cantor method and are in the units of the number of amino acid substitutions per site. The protein EMCL318 retrieved highlighted in red.

For comparison, the predicted amino acid sequences of the F proteins from a number of the members of the *Microviridae* were compared using CLC Sequence Viewer 7 (QIAGEN Aarhus A/S, Aarhus, Denmark). As has been observed previously (e.g., [21]), the *Microviridae* cluster into three groups; ΦX174-like, α3-like, and G4-like. Using either the F protein (Figure 4) or the A protein (data not shown) for alignment, the three clades are clearly visible with WA14, ID18, and EMCL318 forming a distinct cluster within the G4 clade. The similarity between WA14 and ID18 is not surprising since they were isolated ~16 Km apart. However, the remarkable sequence identity between EMCL318 and WA14/ID18 is a bit surprising since they were isolated more than 700 Km away from each other. It is possible, due to this homology, that WA14 and ID18 are also able to infect MDR strains were *E*. *coli* (New Delhi strain) 15-318. These studies are underway since, if there are differences among these phages with regard to host strain, specificity would be due to a very small number of amino acid differences.

## 3. Discussion

There is a growing need for alternative, safe and effective treatment of antibiotic-resistant Gram-negative *Enterobacteriaceae*, particularly *Escherichia coli* [22]. *E. coli* is the most common enteric bacterium that causes nosocomial and community infections [22,23]. There is currently a great deal of interest in whether phages can be used to fight antibiotic-resistant bacterial infections in an effective and specific manner. The purpose of this study was to screen diverse water samples in British Columbia, Canada, and to isolate and characterize a phage that can kill clinical multi-drug-resistant *E. coli*.

Sewage from treatment centers is a rich source of phages [24,25,26]. In comparison to the sewage water samples that contain human excrement consisting of gut microbiota and potential pathogens [27], extreme habitat water and soil samples collected from Bisaro Anima Cave, alkali ponds, and soil samples in our study might not be necessarily exposed to the same species and strains of bacteria associated with humans. Although the presence of *E. coli* in alkali ponds and soil samples has been reported in previous studies [28,29], phages present in these samples might not be specific to the MDR *E. coli* strains used in this study.

These studies describe the isolation of a bacteriophage, designated as EMCL318, from a Kamloops sewage water sample. The EMCL318 phage morphology, as determined by cryo-EM electron microscopy, was found to be an icosahedral particle with features common to the *Microviridae*. *Microviridae* family has been designated as one of the most commonly retrieved ssDNA virus families [30,31]. Interestingly, the *Microviridae* were found to exclusively infect the *Enterobacteria* [18]. Noteworthy, most of the earlier studies with bacteriophages concentrated on the double-stranded DNA (dsDNA) phages [32,33,34,35] while single-stranded DNA (ssDNA) viruses were largely ignored.

SDS-PAGE (Figure 2) and mass spectrometry analysis of purified virion EMCL318 demonstrated that a major component of the phage sample was likely an F protein from G4 phage. A cryo-EM structure (Figure 3) was determined found to be consistent with the structure of a G4 phage that is in the same subfamily with ΦX174 [36]. Finally, ssDNA was isolated from the purified EMCL318 phage, sequenced, and found to be nearly identical to the G4 phages: WA14 (99% identity) and ID18 (98% identity). WA14 was retrieved from Sewage Treatment Plant, Pullman, Washington in November 2001 and ID18 was isolated from Waste Water Treatment Plant, Moscow ID in June 2002 [21]. Studies by Yang et al. 2011 is of particular interest since they genetically manipulated the G4 sequence to chemically synthesize the phage. The infectivity of this synthetic bacteriophage G4 was compared to the wild type G4 and found to be able to infect *Escherichia coli*. This demonstrated the potential use of synthetic biology as a tool in fighting infections [37].

The increased occurrence and severity of the MDR bacterial infections have created a great demand for novel bactericidal approaches. The implication of the self-amplifying ‘drugs’, i.e., bacteriophages, that specifically target and kill bacteria [17,38,39] were thought to be potential therapeutic measures against these MDR strains. This bacteriophage, EMCL318, might prove to be an attractive choice and could be developed as an alternative treatment for multi-drug-resistant *E. coli* infection in human medicine and other application. Interestingly, EMCL318 shows selectivity towards *E*. *coli* 318 MDR strains compared to the *E*. *coli* regular, antibiotic sensitive strain (Figure 1). Similar to these results, a previous study reported isolation of lytic phage, OMKO1, from natural sources, specifically infected 2 MDR *Pseudomonas aeruginosa* strains (PA01 and PA14). However, infectivity of OMKO1 in the sensitive *P. aeruginosa* strain was not determined [9]. The distinguishable intra-species specificities of phages towards bacteria was also reported in a previous study [40]. In conclusion, these results demonstrate that EMCL318 has potential as a novel antibacterial agent against the MDR strains of *E*. *coli.* However, its characteristics, such as host range, heat tolerance, pH stability, one step growth, and its activity comparing to other G4 phages should be further studied.

## 4. Materials and Methods

### 4.1. Sample Collections

Water samples were collected from different areas in British Columbia, Canada. These water samples were collected from the Kamloops Wastewater Treatment Center; Domtar (A local pulp mill), Kamloops; the Pacific Ocean, Bamfield; Bisaro Anima Cave, Fernie, and alkali ponds, Kamloops. All samples were stored at 4 °C until used.

### 4.2. Processing of Water Samples

The collected water samples containing abundant particulates and debris were initially pressure-filtered through 0.45 μM sterile syringe filter (VWR International, Mississauga, ON, Canada), followed by filtration through 0.22 μM sterile syringe filter (VWR International, Mississauga, Canada). The samples devoid of visible debris were directly filtered through 0.22 μM sterile syringe filter.

### 4.3. Host Microbes for Bacteriophage Screening

The non-resistant and MDR *E. coli* strains were used to screen the bacteriophages. The clinical MDR strains were *E*. *coli* (New Delhi strain) 15-318, *E. coli* (NDM type carbapenemase) 15-102, and *E. coli* (oxa48 type carbapenemase) 15-124. The MDR *E*. *coli* 15-102, 15-124, 15-318 strains were provided by LifeLabs, Canada. All three isolates possess resistance to ampicillin, amoxicillin/clavulanic acid, piperacillin/tazobactam, cefalotin, cefazolin, cefoxitin, cefixime, ceftazidime, ceftriaxone, ertapenem, meropenem, amikacin, gentamicin, tobramycin, ciprofloxacin, tetracyclin, nitrofurantoin, trimethoprim/sulfamethoxazole. These clinical isolates were unlikely to possess verotoxin as they were not isolated from diarrheagenic source (personal communication). These isolates came from another type of samples from patients (personal communication). The non-resistant *E*. *coli* strain K12 was available at the Thompson Rivers University, Kamloops, Canada.

### 4.4. Isolation of Bacteriophages

The isolation of the bacteriophage was performed using methods similar to an earlier study [31]. The host bacteria strains were inoculated in nutrient broth (Hardy Diagnostics, Santa Maria, CA, USA) and Sabouraud dextrose broth (HiMedia Laboratories Pvt. Ltd., Nashik, India) media respectively. The bacterial cultures were incubated overnight at 37 °C and mixed at a cell concentration of 10^6^ cells ml^−1^ (approximately) with 12 mL of samples and left on the bench for incubation for 10 min at room temperature (20–22 °C). Following the incubation, the mixture was added to 60 mL of nutrient molten agar and poured into 150 mm Petri-plates for solidification followed by an incubation at 37 °C until the visible plaques (clear zone) were observed.

### 4.5. Initial Purification and Identification of the Phage

Initially, the phage was plaque purified using the 15-318 strain of *E. coli*, at 37 °C. When the titer was not improved from passaging in liquid culture, the incubation was moved to ambient temperature and the phage and cells were shaken for several days. Large 2 l cultures were then grown in a similar manner. After two days, the cells were removed by centrifugation for 30 min at 5000× g. The supernatant was then concentrated to 150 mL using a Pellicon 2 (Millipore) tangential flow concentrator with a 300 kD cutoff. The material was then concentrated by ultracentrifugation at 450,000 rpm for 2.5 h using a 50.2TI rotor (Beckman). The pellets were suspended in 2 mL of PBS and then purified using 7.5–45% linear sucrose gradients using 12.5 mL Ultra-clear (Beckman) tubes and centrifugation at 30,000 rpm in a SW41 rotor (Beckman) for 2 h. The gradient was then collected using an ISCO (Teledyne) fractionator, collecting from the top. While there was a large gradient of optical density extending from the top of the gradient, there was a small peak approximately midway down the tube. Plaque assays demonstrated that this contained the highest viral titer. These fractions were pooled and analyzed by both negative stain electron microscopy (EM) and SDS-PAGE. EM demonstrated that there were particles present and the SDS-PAGE showed several major bands. The two strongest bands were submitted to the UTMB Mass Spectroscopy Core (see Methods below) and the largest band was identified as the F protein from the G4 phage.

With the identification of the phage being G4, several changes were made in the growth and purification procedures. The LB medium was adjusted to include 25 mM MgCl_2_ to improve lysis and titer. The growth conditions and use of the tangential flow concentrator was as described above. However, to the 150 mL of concentrated phage solution, 0.5 M stock of EDTA (pH 8.0) was added to yield a final concentration of 25 mM and powdered *N*-lauroylsarcosine sodium salt was added to a final concentration of 1%. These additions did not affect the yield but removed most of the cellular debris.

### 4.6. Mass Spectroscopy

Isolated SDS-PAGE gel plugs were subjected to proteolysis by proteome grade trypsin (Sigma Aldrich, St. Louis, MO, USA) at pH 8, 37 °C, overnight before analysis by mass spectrometry. The peptide mix was desalted using C18 ZipTips (Millipore, Burlington, MA, USA) and 1 µL of this solution was combined with 1 µL of a 3 mg^−1^mL α-cyano-4-hydroxycinnamic acid (60% acetonitrile, 1 mM ammonium diphosphate) and spotted onto MALDI targets. All MALDI-MS experiments were performed using a 5800 MALDI-TOF/TOF (Applied Biosystems, Foster City, CA, USA). The MS data were acquired using the reflectron detector in positive mode (700–4500 Da, 1900 Da focus mass) using 300 laser shots (50 shots per sub-spectrum). Collision-induced dissociation tandem MS spectra were acquired using 1 kV of collision energy. All MS and MS/MS data were searched against the UniProt protein sequence database using Protein Pilot (Applied Biosystems, Foster City, CA, USA) software. The database search parameters used for Mascot (Matrix Science, London, UK) were the following: mass tolerance: 0.5 Da, taxonomy: phage and *E. coli*, enzyme: trypsin, missed cleavages: 1, and variable modifications: oxidation (M), deamidation (N, Q).

### 4.7. Electron Microscopy

Samples of purified phage were initially analyzed using negative stain. Purified concentrated G4 bacteriophage was negatively stained with 2% uranyl acetate in water on 200 mesh carbon grids (CF-200-Cu, Electron Microscopy Sciences, Hatfield, PA, USA) and imaged using JEOL 2100 electron microscope with Lab_6_ electron gun. Images were acquired at 30,000× indicated magnification with a 4k × 4k slow-scan CCD camera (UltraScan 895, GATAN, Inc., Pleasanton, CA, USA). The pixel size was 3.7 Å on specimen scale. Virus particles were ~300Å in diameter.

For cryo-EM analysis, the phage was vitrified as previously described [41] on carbon holey film (C-flat™, Protochips, Raleigh, NC, USA) grids with EM-GP cryo-plunger (Leica Microsystems Inc. Buffalo Grove, IL, USA). Imaging was performed at 40,000× magnification with ~20 electrons/Å^2^ dose (pixel size 2.8 Å on the specimen scale). Images used for reconstructions had defocus values of 0.74–5.7 µm.

EMAN2 [42] was used for image processing. G4 particles were boxed out from images and the Contrast Transfer Function parameters were determined using EMAN2. Subsequent processing included particle alignment, angular orientation determination and 3D reconstruction.

### 4.8. Nucleotide Sequencing

DNA was purified from the purified phage using the modified protocol for M13 DNA purification from Qiagen. In brief, the phage was precipitated by adding 7 µL of MP buffer (3.3 g citric acid + 3 mL water) to 0.7 mL of purified phage. This solution was allowed to incubate at 24 °C for 2–10 min. This solution was added to the QIAprep spin column and centrifuged for 15 s at 8000 rpm. To the column, 0.7 mL of buffer PB buffer (5.0 M guanidinium hydrochloride +30% isopropanol) was added and spun for 15 s at 8000 rpm. Another 0.7 mL of PB buffer was added to the column, allowed to incubate for 1 min at room temperature to lyse the phage, and then centrifuged for 15 s at 8000 rpm. 0.7 mL of PE buffer (10 mm Tris, pH 7.5 + 80% ethanol) was added to the column and centrifuged as before. The column was washed again with PE buffer and then centrifuged again to remove residual PE buffer. 100 µL of EB buffer (10 mM Tris, pH 8.5) was added to the column, incubated for 10 min, and the DNA was collected with a 30 s centrifugation at 8000 rpm. The phage DNA was then analyzed with a 1% agarose gel and appeared as a single band ~5.5 kb in size. Sanger-based DNA sequencing was performed using a cycle sequencing approach that utilizes the incorporation of fluorescent-labeled dideeoxynucleotides. Amplified products were analyzed using capillary electrophoresis on an Applied Biosystems 3130 Genetic Analyzer. The phage is available upon request.
